# High incidence of (ultra)low oesophageal temperatures during cryoballoon pulmonary vein isolation for atrial fibrillation

**DOI:** 10.1007/s12471-020-01493-z

**Published:** 2020-11-10

**Authors:** M. M. D. Molenaar, T. Hesselink, M. F. Scholten, K. Kraaier, D. E. Bouman, M. Brusse-Keizer, Y. J. Stevenhagen, P. F. H. M. van Dessel, B. ten Haken, J. G. Grandjean, J. M. van Opstal

**Affiliations:** 1 Thoraxcenter Twente, Medisch Spectrum Twente, Enschede, The Netherlands; 2 Department of Magnetic Detection and Interventions, University of Twente, Enschede, The Netherlands; 3 Radiology Department, Medisch Spectrum Twente, Enschede, The Netherlands; 4 Medical School Twente, Medisch Spectrum Twente, Enschede, The Netherlands

**Keywords:** Atrial fibrillation, Pulmonary vein isolation, Safety, Oesophageal temperature, Computed tomography

## Abstract

**Background:**

Low oesophageal temperatures (OTs) during cryoballoon pulmonary vein isolation (PVI) have been associated with complications. This study assessed the incidence of low OT in clinical practice during cryoballoon PVI and verified possible predictive values for low OT.

**Methods:**

Consecutive patients who underwent PVI using the second-generation cryoballoon were retrospectively included. The distance from the oesophagus to the different pulmonary veins (PVs) (OP distance), body mass index (BMI), sex, age, balloon temperature and application time were studied as potential predictors of low OTs. Computed tomography was performed before the procedure to determine the OP distance. OT was measured using an oesophageal temperature probe. Applications were ended prematurely if the OT reached <16 °C. Low and ultralow OT were defined as OT <20 and <16 °C respectively.

**Results:**

Two hundred and four patients were included. Low OT was observed in 54 patients (26%) and 27 patients (13%) reached ultralow OTs. OP distance was the only predictor of low OTs after multivariate analysis. A cut-off value of 19 mm showed 96.2% sensitivity and 37.8% specificity in predicting low OTs. No clinically relevant relation was found between low OTs and BMI, age, sex, balloon temperature or application duration.

**Conclusions:**

The incidence of low OT was 26% for cryoballoon PVI. OP distance was the only predictor of low OTs. Since an OP distance <19 mm was present in all patients in at least one PV, we recommend routine OT measurement during PVI cryoballoon therapy to prevent oesophagus-related complications.

## What’s new?

- Second-generation cryoballoon pulmonary vein isolation results in an oesophageal temperature (OT) <20 °C in 26% of patients. Half of them even showed an OT <16 °C.
- Oesophagus to pulmonary vein (OP) distance was identified as the only predictor of low OTs.
- No clinically relevant relation was found between low OTs and body mass index, balloon temperature or application duration.
- A cut-off value for OP distance of 19 mm showed a 96.2% sensitivity and a 37.8% specificity for predicting low OTs.

## Background

Pulmonary vein isolation (PVI) is an established treatment option for atrial fibrillation (AF). Cryoballoon catheters are increasingly used to perform PVI as a successful alternative to point-by-point radiofrequency ablation.

However, cryothermal energy can reach the surrounding tissues, potentially causing collateral damage. Oesophageal lesions, atrio-oesophageal fistulae and vagal nerve injury, resulting in gastroparesis, have been related to low oesophageal temperatures (OTs) [1–7]. OTs ≤12 °C have shown a 100% sensitivity and 92% specificity in predicting the formation of gastro-oesophageal lesions [3]. Interruption of cryoablation at an OT of 15 °C has been associated with reduced incidence of oesophageal injury [8].

In clinical practice low OTs occur on a regular basis. Finding predictive parameters for low OTs would enable precautions to be taken to prevent collateral damage. This study aimed to retrospectively assess the incidence of low and ultralow OT, defined as OT <20 °C respectively <16 °C, in clinical practice during regular PVI using the second-generation cryoballoon. Furthermore, we hypothesised that predictive parameters for (ultra)low temperatures could be found in the anatomical position of the oesophagus in relation to the different pulmonary veins (PVs), body mass index (BMI), age, sex, balloon temperature and/or application time.

## Methods

### Patients

Patients accepted for cryoballoon PVI according to current international guidelines at the Medisch Spectrum Twente (Enschede, The Netherlands) were included in this single-centre retrospective study [9]. The need for informed consent was waived by the medical ethical committee and this study complies with the Declaration of Helsinki.

### Cardiac CT PVI protocol

Pre-PVI image acquisition by cardiac computed tomography (CT) angiography was performed on a Toshiba Aquilion 64 CT scanner (Toshiba Medical Systems, Tokyo, Japan). If possible, CT scanning was synchronised with the electrocardiogram. Parameters were field of view 500 mm, 120 kV, 300 mAs (automatically adjusted to the cardiac cycle typically at 75%). Image acquisition was performed using a 0.5 mm slice thickness and 0.3 mm slice interval.

A bolus of 80 ml iodinated contrast agent (Visipaque, GE Healthcare, Chicago, IL, USA, 320 mg I/ml) was injected into the antecubital vein at 3 ml/s followed by saline chaser of 20 ml. Bolus tracking was performed until a region of interest of >170 Hounsfield units was measured in the left atrium.

### PVI procedure

**Fig. 1:**
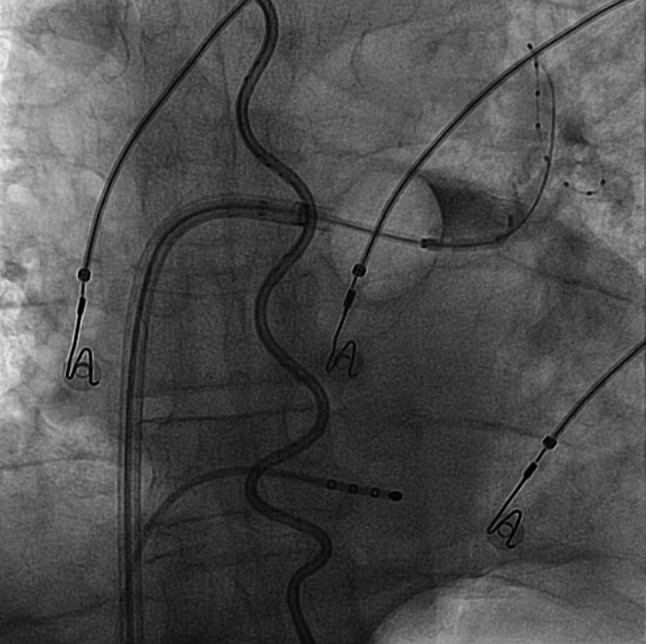
Position of the thermoprobe (S-shaped) in the oesophagus with cryoballoon positioned at the antrum of the left superior pulmonary vein with the lasso catheter inside this pulmonary vein and contrast dye being injected into the pulmonary vein (*right upper quadrant*). The stimulation catheter is positioned in the coronary sinus

All procedures were performed with the patient under general anaesthesia with continuous arterial blood pressure monitoring. Heparin was administered to achieve an activated clotting time of >300 s during the procedure. A temperature probe was inserted into the oesophagus under fluoroscopic guidance. In the first 79 patients a temperature probe with three thermocouples separated by 10 mm (SensiTherm, St Jude Medical Inc., Saint Paul, MN, USA) was used; the position of this temperature probe was adjusted to the fluoroscopic position of the balloon during each application. In the last 126 patients an S‑shaped temperature probe with 12 electrically insulated temperature sensors (CIRCA S‑CATH, CIRCA Scientific, Englewood, CO, USA) was used (Fig. 1). The use of a 23- or 28-mm balloon (Arctic Front Advance, Medtronic Inc., Minneapolis, MN, USA) was based on the PV diameters. Optimal positioning of the balloon was achieved by integrating the available CT images in a 3D system (Philips, The Netherlands) or by angiography of the PVs [10].

During cryoablation of the right-sided PVs the right phrenic nerve was continuously stimulated by pacing from the superior cava vein or the right subclavian vein. If diminished diaphragm excursion was observed during cryotherapy, the application was stopped immediately using the double-stop technique [11]. If the OT reached <16 °C, the application was stopped prematurely [8]. No subsequent application was initiated until the OT reached >30 °C again.

Initially, two applications were performed and the target application time exceeded 240 s in only five cases. Successful isolation of a PV was proven by entrance and exit block. If isolation was unsuccessful after the initial two applications, ablation was continued until complete isolation was achieved. At the end of the procedure, possible dormant conduction was evaluated using adenosine.

### CT measurements

**Fig. 2:**
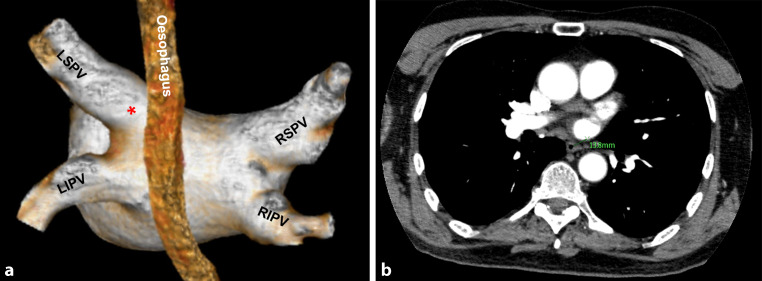
**a** Shortest oesophagus to pulmonary vein distance measurement when we first looked at the CT images. The origin of the os, identified as the indentation in the posterior wall, caused by the angulation of the pulmonary vein compared to the atrial wall. The angulation between the pulmonary vein and the atrium can be well distinguished in this 3D view. The distance from the identified angulation (*) to the oesophagus in the axial plane represents the shortest oesophagus to pulmonary vein distance. *LSPV* left superior pulmonary vein, *RSPV* right superior pulmonary vein, *LIPV* left inferior pulmonary vein, *RIPV* right inferior pulmonary vein. **b** This distance was measured

For this study the CT images acquired pre-PVI were used to measure the shortest distance between the oesophagus and the os of every PV (OP distance). To measure this OP distance, the origin of the os was identified as the indentation in the posterior wall, caused by the angulation of the PV compared to the atrial wall (Fig. 2a). Since the oesophagus runs perpendicular to the axial plane, the distance from the identified angulation to the oesophagus in the axial plane represents the shortest OP distance. This distance was measured (Fig. 2b).

### Statistical analysis

Low OT was defined as OT <20 °C and ultralow OT as OT <16 °C. Continuous variables are reported as mean with SD (normally distributed) or as median with interquartile range (IQR) (non-parametric data). Categorical variables are displayed as numbers. Comparison of baseline characteristics between patients with and without low OT was performed using an independent *t*-test or Mann-Whitney U test as appropriate for continuous variables. For comparison of categorical variables the chi-square or Fisher exact test was used.

Correlation coefficients were calculated (Pearson or Spearman correlation tests) to test the association of the potential predictors, OP distance, BMI, age, sex, balloon temperature and application time, with the lowest OT. A correlation coefficient <0.25 was considered not clinically relevant. Univariate logistic regression analyses were performed to select predictors for low OT. All variables with *p* < 0.10 were entered in a multivariate logistic regression analysis. Subsequently variables with the highest *p*-values were deleted until the fit of the model decreased significantly (based on −2 log likelihood). A two-sided *p-*value of <0.05 was considered statistically significant. Statistical analyses were performed with SPSS v.22.

## Results

### Incidence

**Fig. 3:**
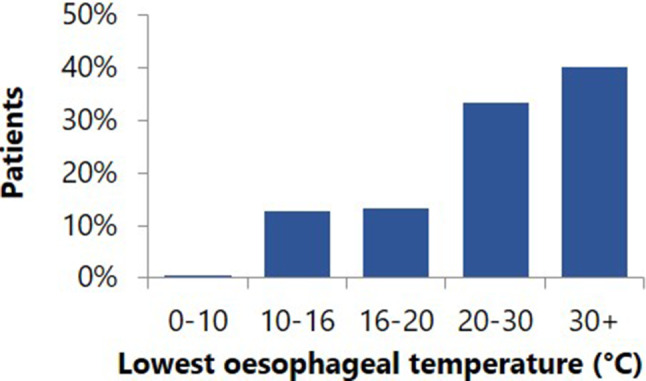
Lowest oesophageal temperatures during pulmonary vein isolation

Two hundred and four patients were included. Baseline characteristics of the study cohort are shown in Tab. 1. Median time between CT acquisition and PVI was 66 days (IQR 38–99). Fig. 3 shows the distribution of OTs reached. In 54 patients (26%), 2 with the 23-mm balloon, OTs below 20 °C were reached. In 27 patients (13%) the lowest OT was <16 °C and in 1 patient the OT dropped below 10 °C (7.6 °C). In 23 patients applications were prematurely stopped because of (ultra)low OT. In 4 patients with low OTs, applications were not prematurely terminated. Low OTs were reached after the regular application time had been completed, due to the latency effect. There were no significant differences in baseline characteristics between the (ultra)low OT group and the group without low OT. In all but 5 patients the 28-mm balloon was used, in 6 patients both the 28-mm and the 23-mm balloon were used. Low OTs were found in 8% of the PVs in which the SensiTherm was used and in 9% of the PVs in which the Circa S probe was used (*p* = 0.67). In 168 patients a CT image was available for measurement of the OP distance. Lowest OTs occurred in the left inferior (LI) and right inferior (RI) PVs (Tab. 2). Median OP distance was 8.7 (5.4–12.3) mm for left superior PV, 7.6 (4.7–11.8) mm for the LIPV, 29.3 (23.7–34.7) mm for the right superior PV and 17.0 (11.8–22.7) mm for the RIPV. During regular clinical follow-up for 1 year no clinical consequences were demonstrated within the (ultra)low OT group.

**Table 1:** Baseline characteristics (*n* = 204)

Parameter	No low OT (*n* = 148)	Low OT (*n* = 56)
Age (years)	56 ± 11	58 ± 12
Gender: male, *n* (%)	105 (71)	36 (64)
LA diameter, *n* (%)^a^		
– Normal	126 (85)	46 (82)
– Mildly dilated	17 (12)	7 (13)
– Moderately dilated	3 (2)	0 (0)
– Severely dilated	1 (1)	1 (2)
– Unknown	1 (1)	2 (4)
Medical history, *n* (%)		
– Hypertension	45 (30)	12 (21)
– Diabetes mellitus	5 (3)	1 (2)
– Coronary artery disease	12 (8)	4 (7)
– Stroke	2 (1)	0 (0)

*OT* oesophageal temperature

^a^Left atrial (*LA*) diameter ranges for cc/m^2^ as in Lang et al. 2015 [35]. Normal 16-34 cc/m^2^, mildly dilated 35-41 cc/m^2^, moderately dilated 42-48 cc/m^2^, severely dilated >48 cc/m^2^

**Table 2:** Number of (ultra)low oesophageal temperatures by pulmonary vein (*PV*) in which these occurred

PV	Temperature (°C)		
	20–16	16–10	10–0
LSPV	0	1	0
LIPV	18	16	1
RSPV	2	0	0
RIPV	7	9	0

*LS* left superior, *LI* left inferior, *RS* right superior, *RI* right inferior

### Predictors

**Fig. 4:**
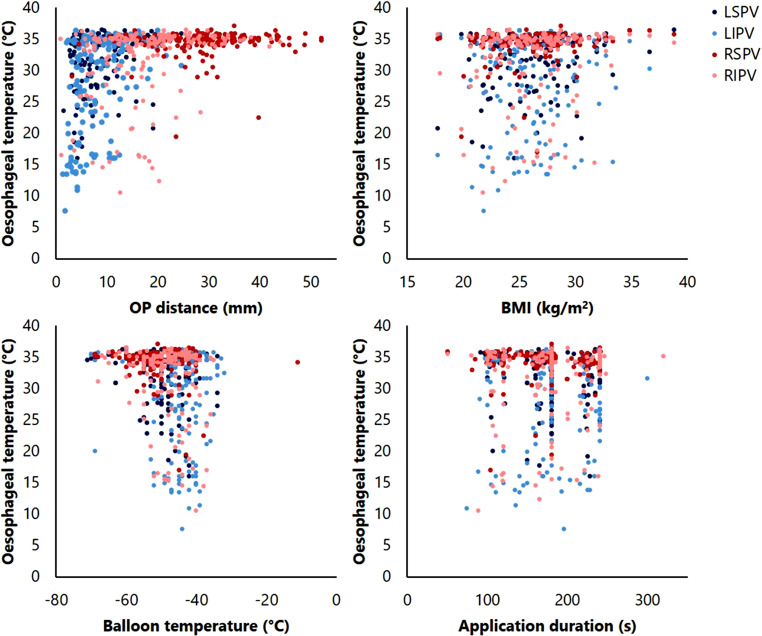
Relation of lowest oesophageal temperature with corresponding distance from oesophagus to pulmonary vein (*OP distance*), body mass index (*BMI*), balloon temperature and application duration. *LSPV* left superior pulmonary vein, *RSPV* right superior pulmonary vein, *LIPV* left inferior pulmonary vein, *RIPV* right inferior pulmonary vein

No clinically relevant correlation with OT was found for BMI, age, sex, balloon temperature or application time. A significant and clinically relevant correlation of 0.405 was found for OP distance and OT (Fig. 4). Univariate logistic regression analysis showed that low OTs (<16%) were negatively associated with OP distance (OR 0.85; 95% CI 0.80–0.90; *p* < 0.001), and positively associated with balloon temperature (OR 1.08; 95% CI 1.03–1.12; *p* = 0.001). BMI (OR 0.92; 95% CI 0.85–1.01; *p* = 0.072), age (OR 1.01; 95% CI 0.99–1.04; *p* = 0.41), male gender (OR 1.56; 95% CI 0.87–2.77; *p* = 0.14) and application time (OR 0.99; 95% CI 0.99–1.00; *p* = 0.075) were not independently associated. OP distance, balloon temperature, BMI and application time were included in the multivariate analysis based on the level of significance.

In multivariate logistic regression analysis, OP distance (OR 0.84; 95% CI 0.791–0.895; *p* < 0.000) and application time (OR 0.99; 95% CI 0.985–0.999; *p* = 0.020) were the only independent predictors of low OTs. Since the application time was shortened in the group of patients reaching ultralow OTs, a sensitivity analysis was performed and ultralow OTs were excluded. The sensitivity analysis showed that there was no predicting effect of application time. Therefore, the OP distance was the single independent predictor of low OTs. Assessment of the sensitivity and specificity was performed for several cut-off values (Tab. 3). Since this parameter would be used for prevention the focus should be on a high sensitivity, preferably >95%. Therefore we selected a cut-off value of 19 mm for OP distance. This showed a 96.2% sensitivity and 37.8% specificity in predicting low OTs (Tab. 3 and 4). An OP distance <19 mm was present in all patients in at least one PV.

**Table 3:** Sensitivity and specificity for oesophagus to pulmonary vein (*OP*) distance cut-off values

OP distance cut-off (mm)	Sensitivity (%)	Specificity (%)
15	86.5	47.8
18	92.3	40.3
19	96.2	37.8
20	96.2	35.2
23	98.1	28.8

**Table 4:** Occurrence of low oesophageal temperature (*OT*) per pulmonary vein (PV) for oesophagus to PV (*OP*) distances smaller and greater than 20 mm

	Oesophageal temperature	
OP distance	Low OT	No low OT
<20 mm	50	376
>20 mm	2	204

## Discussion

In our study population second-generation cryoballoon PVI showed low OTs, below 20 °C, in 54 patients (26%). In half of them ultralow OTs, below 16 °C, were measured despite the cessation of cryotherapy at 16 °C. Multivariate analysis identified OP distance as the only predictor of low OTs. A cut-off value of 19 mm showed a 96.2% sensitivity and a 37.8% specificity for predicting low OTs. No clinically relevant relation was found between low OTs and BMI, age, sex, balloon temperature or application duration.

With the introduction of the second-generation balloon the success of PVI increased [12, 13]. However the potential for inadvertent collateral injury has increased too, since cryothermal energy disperses into the surrounding tissues. This involves damage to the oesophagus (oesophageal lesions and even fistulas), as well as damage to the vagal and phrenic nerves, but bronchial effects also have been described [1–7, 14, 15].

Several studies performing systematic post-procedural oesophagoscopy post-PVI have demonstrated a high incidence of oesophageal lesions (18.8%) [3, 8, 16–18]. A high incidence of gastric motility disturbance, which can result in gastroparesis, has been described too (17.3%) [6, 7, 19, 20]. This relates to the vagus nerve, which is situated close to the heart and spreads out on the oesophagus in a web-like structure. It innervates the stomach, to provide gastric motility, and controls the pyloric sphincter.

In our study the OP distance, as determined on CT images acquired pre-PVI, was the single predictor of low OTs, with a cut-off value of 20.0 mm showing a high sensitivity. However, in every patient at least one of the PVs showed an OP distance <19 mm, which implies that a temperature probe should be routinely used. Unfortunately, the specificity of this predictor was fairly low. An explanation could be the variability of the oesophageal position over time. Research in that field, in a small numbers of subjects, showed evidence for a fixed as well as a variable position [21–23].

The relation between OP distance and OT has only been shown for radiofrequency PVI and ultrasound balloon PVI so far, but seems trivial [24, 25]. Martinek et al. showed oesophagus to left atrium distance to be the single predictor of oesophageal ulcerations in radiofrequency PVI [26]. Recently, Miyazaki et al. assessed the relation between OP distance and gastric hypomotility in cryoballoon PVI and found a cut-off value of 18.2 mm for the RIPV (88.1% sensitivity 77.8% specificity) [19]. It was also suggested that the anatomical relation between PVs and the aorta may identify patients prone to have low OTs [19, 27].

Another explanation could be that the pressure applied to the PV, when occluding it using the balloon, changes the relationship between the LA and the oesophagus. In our study, low and ultralow OT occurred predominantly in the inferior PVs (Tab. 2), which can be explained by the anatomical course of the PVs. As the inferior PVs run posteriorly, the balloon is being pushed towards the oesophagus in these PVs.

OT guidance of cryoballoon PVI has shown a significant reduction of thermal oesophageal lesions and interrupting applications at an OT of 15 °C has been suggested to prevent oesophageal injury [8]. Our study shows that low OT is found in a considerable number of patients during cryoballoon PVI, but temperature probes are neither routinely used in the Netherlands nor recommended. This is possibly due to the extra costs involved and the lack of an apparent increase in significant complications in hospitals not using the probe. However, without oesophageal monitoring low temperatures remain unnoticed. Since symptoms of oesophageal and vagal nerve damage are not usually linked to a cardiac procedure, these complications are not always attributed to this procedure and might be under-reported. Furthermore, the incidence of very serious complications (atrio-oesophageal fistulae and complete gastroparesis) is low but devastating [28–30].

In the first 79 patients a straight temperature probe with three thermocouples (SensiTherm) was used. A concern when using this probe is the possible underestimation of the OT. It can be positioned contralateral to the side where the energy is applied or be floating free in the oesophagus [31, 32]. Furthermore, the coldest temperature could occur in between the thermocouples. During the study period the most recent thermoprobe (CIRCA S‑CATH) was introduced and some of these disadvantages seem to have been eliminated ([33]; Fig. 1). It contains 12 electrically insulated temperature sensors which are positioned with smaller spacing. Due to the S‑shape of the probe it touches the oesophagus walls and covers the whole length of the oesophagus in relation to the PVs, which obviates the need to reposition the probe during the procedure. Therefore we switched to the CIRCA S‑CATH in the remaining 125 patients. The use of two different probes might have had an effect on the recorded OTs. However, no differences between these two groups were found as regards the occurrence of low OTs.

Balloon temperatures are often used as a surrogate metric for OTs and for possible low temperatures in adjacent structures. The absence of a clinically significant correlation between minimum balloon temperature and lowest OT found in our study is in accordance with earlier reports on this subject [3, 8].

Despite the cessation of cryotherapy when the OT reached <16 °C, OTs far below 16 °C were seen. This decline in OT even after termination of the freeze cycle has been reported by Deiss et al. [34]. It is also known as the ‘latency effect’. The maximum latency effect found in that study was a decline of 6.4 °C. In our study an even larger maximum latency effect of 8.4 °C (16 °C to 7.6 °C) was seen, which underlines the importance of this effect when applying safety cut-offs.

## Study limitations

The existence of variability in the oesophageal position is still under debate. Research with small numbers of patients has provided evidence for a fixed as well as a variable position [21–23, 33]. If the oesophageal position indeed has active dynamics, this could have affected our results. If serious migration of the oesophagus between CT image acquisition and the performance of ablation is possible, this could have affected the OP distance and therefore its predictive value.

No endoscopic inspection was performed to assess the possible extent of oesophageal injury. This has been addressed in prior studies, which uniformly demonstrated a relation between low OT and oesophageal lesions [3, 8, 16].

The use of two different probes might have had an effect on the recorded OTs. However, no differences between these two groups were found as regards the occurrence of low OTs. Finally, the retrospective design has to be taken into account.

## Conclusion

Second-generation cryoballoon PVI results in OT <20 °C in 26% of patients. Half of them even showed OT <16 °C. Multivariate analysis identified OP distance as the only predictor of low OTs. No clinically relevant relation was found between low OTs and BMI, age, sex, balloon temperature or application duration. A cut-off value for OP distance of 19 mm showed a 96.2% sensitivity and a 37.8% specificity for predicting low OTs. As an OP distance <19 mm was present in all patients in at least one PV, we recommend routine OT measurement in all patients during cryoballoon therapy to prevent oesophagus-related complications.
